# Supplementary material for: Longitudinal multi-omics study reveals common etiology underlying association between plasma proteome and BMI trajectories in adolescent and young adult twins
Source: BMC Med. 2023 Dec 21;21:508. doi: 10.1186/s12916-023-03198-7 (PMC10740308; doi:10.1186/s12916-023-03198-7)

Supplementary document

Drouard et al.

last update: November 2023

1 Validity of self-reported *versus* measured anthropometric measurements

We wished to investigate the validity of self-reported weight and height measurements in FinnTwin12. Of the 786 FinnTwin12 participants who had blood sampling, 756 had a clinical measure of their weight (kg) and height (cm) in addition to their self-reported weights (kg) and heights (cm). Five median days separated clinical measurements from receipt of self-reported measurements. Thirteen days at most separated weight and height measurements from self-reported measurements for 95% of these participants. Four participants had reported their weight and height more than 1 year before their clinical measurement, and were therefore excluded to improve the assessment of the validity of self-reported measurements.

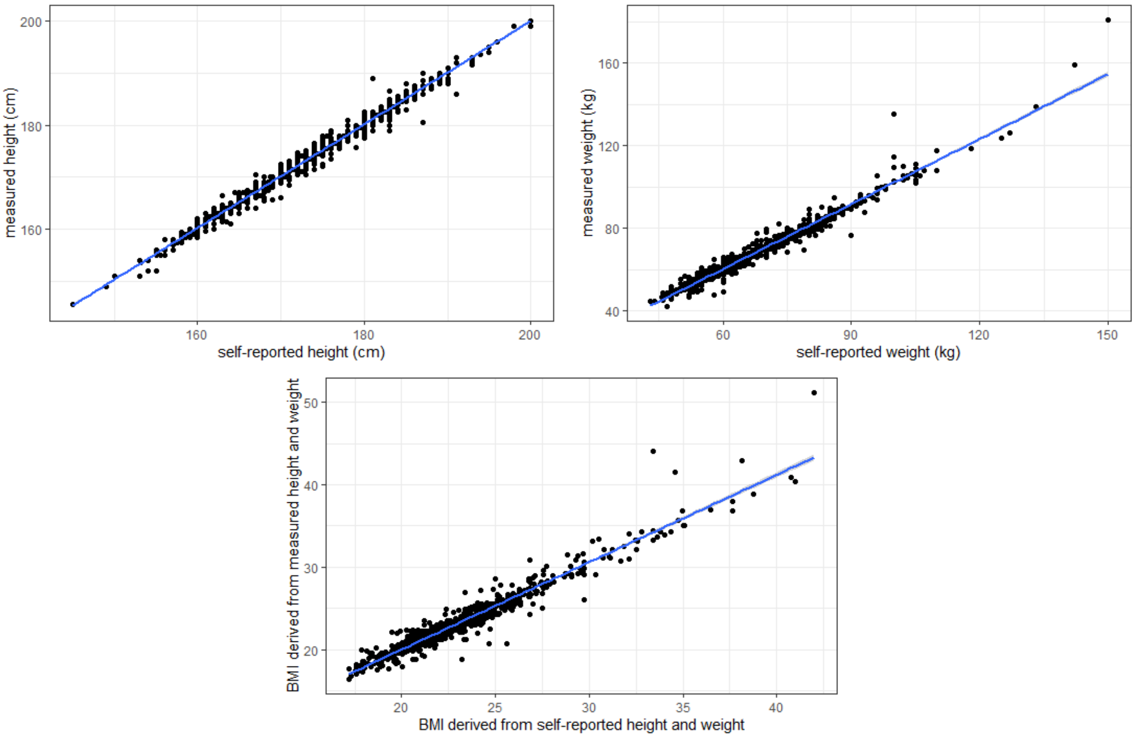

Self-reported height, weight and BMI

		height		weight		BMI	
	N	mean	sd	mean	sd	mean	sd
All	752	171.5	9.5	68.6	14.2	23.2	3.6
Male participants	316	179.5	6.9	77.0	13.2	23.8	3.3
Female participants	436	165.7	6.3	62.5	11.6	22.7	3.8

Measured height, weight and BMI

		height		weight		BMI	
	N	mean	sd	mean	sd	mean	sd
All	752	171.7	9.5	69.4	15.2	23.4	4.0
Male participants	316	179.6	7.1	77.6	14.8	24.0	3.9
Female participants	436	166.0	6.4	63.5	12.5	23.0	4.0

Pearson correlation coefficients were used to compare self-reported and measured height and weight. The body mass index (BMI) derived from the self-reported measurements (kg.m-2) was also compared with the BMI derived from the measured height and weight. Linear regressions were also used to model the relationship between self-reported (x) and measured (y) variables as (Δ).

$$y = \beta_0 + \beta_1 x + \varepsilon \qquad (\Delta)$$

Measured and self-reported height were highly correlated (r=0.99). Measured and self-reported weight also correlated strongly (r=0.98). The BMI derived from the self-reported measurements and the BMI derived from the clinical measurements were strongly correlated (r= 0.97). Linear regressions also showed high agreement between self-reported and measured height, weight, and BMI.

According to linear regressions, there seems to be a great match between self-reported height and measured height. Individuals with high weight tended to slightly underestimate their weight (β_1=1.048). The diagnostic of the residuals shows overall correct quality modeling for height and weight, but some individuals may have inflated the slope coefficient values. The outputs of the linear regressions are therefore to be considered with caution.

	Dependent variable:
	measured height
self-reported height (β_1)	0.994^{***}
	(0.004)
Constant (β_0)	1.167
	(0.734)
Observations	752
R^2	0.986
Adjusted R^2	0.986
Residual Std. Error	1.110 (df = 750)
F Statistic	$54{,}098.020^{***}$ (df = 1; 750)
Note:	*p<0.1; **p<0.05; ***p<0.01

	Dependent variable:
	measured weight
self-reported weight (β_1)	1.048^{***}
	(0.007)
Constant (β_0)	-2.495^{***}
	(0.517)
Observations	752
R^2	0.964
Adjusted R^2	0.964
Residual Std. Error	2.881 (df = 750)
F Statistic	$20{,}169.380^{***}$ (df = 1; 750)
Note:	*p<0.1; **p<0.05; ***p<0.01

	Dependent variable:
	measured BMI
BMI from self-report (β_1)	1.058***
	(0.010)
Constant (β_0)	−1.138***
	(0.237)
Observations	752
R^2	0.936
Adjusted R^2	0.936
Residual Std. Error	1.009 (df = 750)
F Statistic	11,037.050*** (df = 1; 750)
Note:	*p<0.1; **p<0.05; ***p<0.01

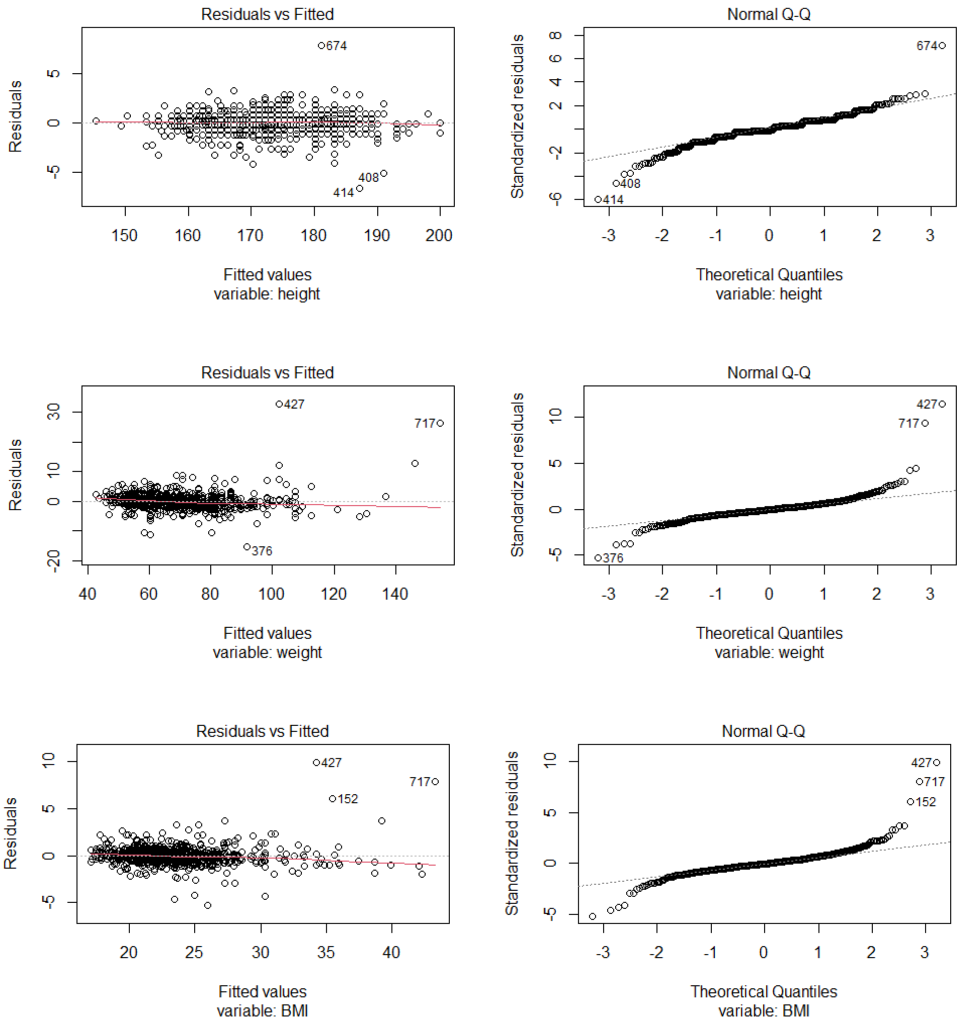

Supplement: Supplementary file 2 — Additional file 2: Supplementary document. Validity of self-reported versus measured anthro-pometric measurements. [file 12916_2023_3198_MOESM2_ESM.pdf]
